# Oral Drug Delivery Systems Based on Ordered Mesoporous Silica Nanoparticles for Modulating the Release of Aprepitant

**DOI:** 10.3390/ijms22041896

**Published:** 2021-02-14

**Authors:** Theodora Christoforidou, Dimitra Giasafaki, Eleftherios G. Andriotis, Nikolaos Bouropoulos, Nikoleta F. Theodoroula, Ioannis S. Vizirianakis, Theodore Steriotis, Georgia Charalambopoulou, Dimitrios G. Fatouros

**Affiliations:** 1 Laboratory of Pharmaceutical Technology, Department of Pharmaceutical Sciences, Aristotle University of Thessaloniki, 54124 Thessaloniki, Greece; dorachristophedc@gmail.com (T.C.); dfatouro@pharm.auth.gr (D.G.F.); 2 National Centre for Scientific Research “Demokritos”, 15341 Athens, Greece; d.giasafaki@inn.demokritos.gr (D.G.); t.steriotis@inn.demokritos.gr (T.S.); 3 Foundation for Research and Technology Hellas, Institute of Chemical Engineering and High Temperature Chemical Processes, 26504 Patras, Greece; nbouro@iceht.forth.gr or; 4 Department of Materials Science, University of Patras, 26504 Patras, Greece; 5 Laboratory of Pharmacology, Department of Pharmaceutical Sciences, Aristotle University of Thessaloniki, 54124 Thessaloniki, Greece; theodorn@pharm.auth.gr (N.F.T.); ivizir@pharm.auth.gr (I.S.V.); 6 Department of Life and Health Sciences, University of Nicosia, CY-1700 Nicosia, Cyprus

**Keywords:** mesoporous materials, poorly soluble drugs, aprepitant, oral delivery

## Abstract

Two different types of ordered mesoporous nanoparticles, namely MCM-41 and MCM-48, with similar pore sizes but different pore connectivity, were loaded with aprepitant via a passive diffusion method. The percentage of the loaded active agent, along with the encapsulation efficiency, was evaluated using High-performance Liquid Chromatography (HPLC) analysis complemented by Thermogravimetric Analysis (TGA). The determination of the pore properties of the mesoporous particles before and after the drug loading revealed the presence of confined aprepitant in the pore structure of the particles, while Powder X-ray Diffractometry(pXRD), Differential Scanning Calorimetry (DSC), and FTIR experiments indicated that the drug is in an amorphous state. The release profiles of the drug from the two different mesoporous materials were studied in various release media and revealed an aprepitant release up to 45% when sink conditions are applied. The cytocompatibility of the silica nanoparticles was assessed in Caco-2 cell monolayers, in the presence and absence of the active agent, suggesting that they can be used as carriers of aprepitant without presenting any toxicity in vitro.

## 1. Introduction

Mesoporous Silica Nanoparticles (MSNs) have been used in a variety of applications ranging from nanoreactors to adsorbents, biosensors, drug delivery systems, gene carriers, phototherapy, and tissue engineering [1]. As drug nanocarriers, MSNs have been widely studied, as they meet key criteria such as biocompatibility, high loading capacity, no premature release, specific targeted delivery, and controlled release of the active ingredients [1,2]. In fact, because of these properties but also due to their uniform and tunable pore size, high surface area, large pore volume, and control over the pore network structure, coupled with significant chemical, temperature, and mechanical stability, they are considered among the most promising materials for drug delivery applications [1,3,4,5,6]. In the current study ordered MSNs are evaluated, for the first time to the best of our knowledge, as potential nanocarriers of aprepitant, a high-affinity antagonist of human substance P/neurokinin 1 (NK1) receptors [7].

**Figure 1:**
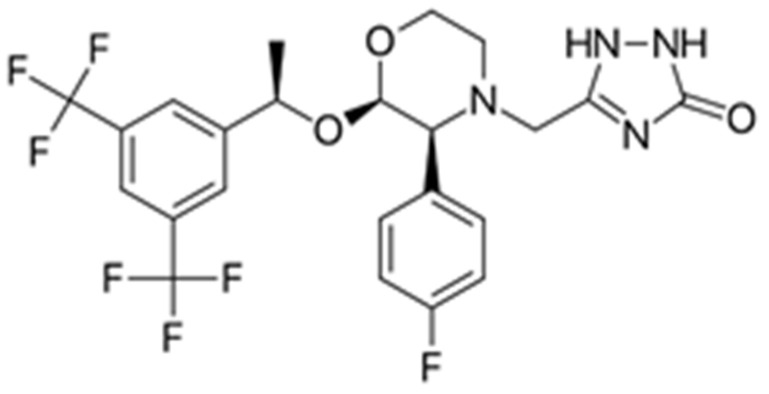
Chemical structure of aprepitant.

Aprepitant (Figure 1) is an orally administrated medication against chemotherapy-induced and postoperative nausea and vomiting. It is characterized as a basic compound, with a pKa value of 9.7 (pH range 2 to 12) [7]. The solubility of this substance is considered very low (3–7 μg/mL) in the pH range of 2–10, while the log *p* value of 4.8 at pH 7.0 suggests high lipophilicity [7]. As an Active Pharmaceutical Ingredient (API) it is classified as “low permeable” and “low soluble” (BCS, Class V) [8], thus requiring relatively high doses [9,10,11,12]. In this context, the development of novel formulations containing aprepitant that may enhance its solubility and bioavailability is considered of great importance for the reduction of the required dose [8,12,13].

A broad range of methods have been proposed for co-formulating aprepitant with different carriers for oral delivery, including micro-emulsions [14,15], self-emulsified drug delivery systems (SEDDS) [16], solid dispersions [17,18,19,20,21], hot-melt extrusion [18], liquisolid formulations [22], and inclusion complexes (cyclodextrins) [13,23].

The present work exploits the particular advantages of MSNs, such as MCM-41 and MCM-48, for the encapsulation and delivery of aprepitant. Such materials (and especially MCM-41) are among those most commonly used for adsorption and release of active ingredients in drug delivery applications [1,24,25,26]. They exhibit a highly ordered mesostructure with cylindrical-like pores, which are organized either in parallel in a 2D hexagonal non-intersecting structure (MCM-41), or in a cubic gyroid minimal surface 3D network (MCM-48) [26]. In this work, MCM-41 and MCM-48 particles were prepared, aiming at a spherical morphology and were loaded with aprepitant via the solvent method (passive diffusion). The structural, thermal, and physical properties of the drug-loaded systems were analyzed and complemented with in vitro drug release studies as well as in vitro cytotoxicity studies, which confirmed the promising performance of the studied MSNs for the effective delivery of aprepitant.

## 2. Results

### 2.1. Scanning Electron Microscopy (SEM)

**Figure 2:**
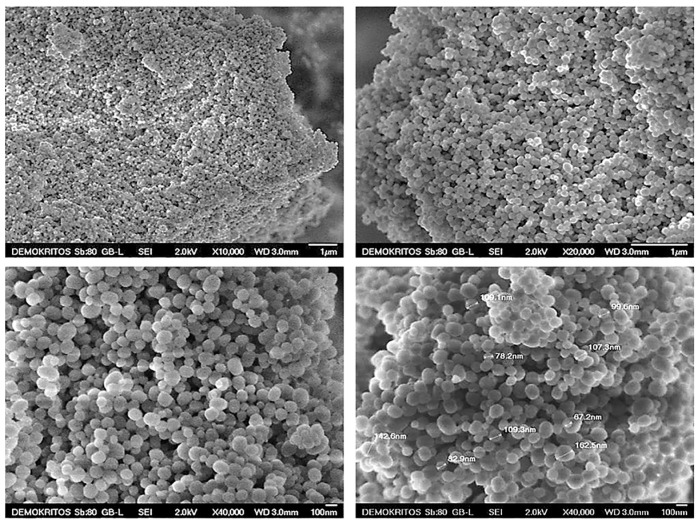
SEM micrographs of the produced MCM-41 particles.

**Figure 3:**
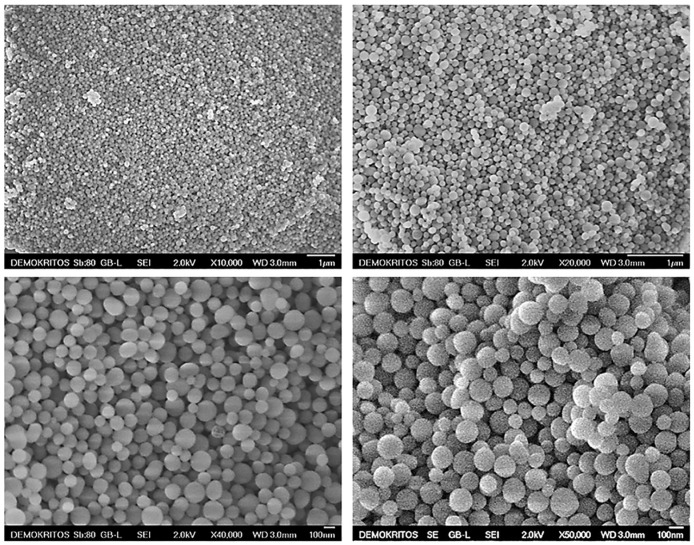
SEM micrographs of the produced MCM-48 particles.

The morphology of the prepared MSN particles was evaluated using SEM analysis. As shown in Figure 2, the produced MCM-41 particles have a quite uniform spherical shape with mean diameters in the range of 80–130 nm. Figure 3 pertains to MCM-48 particles, which also exhibit a spherical morphology, with particle sizes between approximately 100–150 nm. In general, the two types of MSN particles look rather similar, however it may be argued that in the case of MCM-41 more agglomerated and/or fused particles can be observed.

### 2.2. Loading and Encapsulation Efficiency

The drug-loaded MSN nanoparticles were prepared by the passive diffusion method. The amount of aprepitant loaded into the MSNs was determined indirectly by measuring the amount of the drug present in the ethanolic supernatant solution by high-performance liquid chromatography (HPLC); details are provided in Section 4.3. The theoretical drug loading value was calculated equal to 33%, while the individual drug loading and encapsulation efficiency values of the respective MSN particles are summarized in Table 1; details are provided in Section 4.2 and Section 4.4.

**Figure 4:**
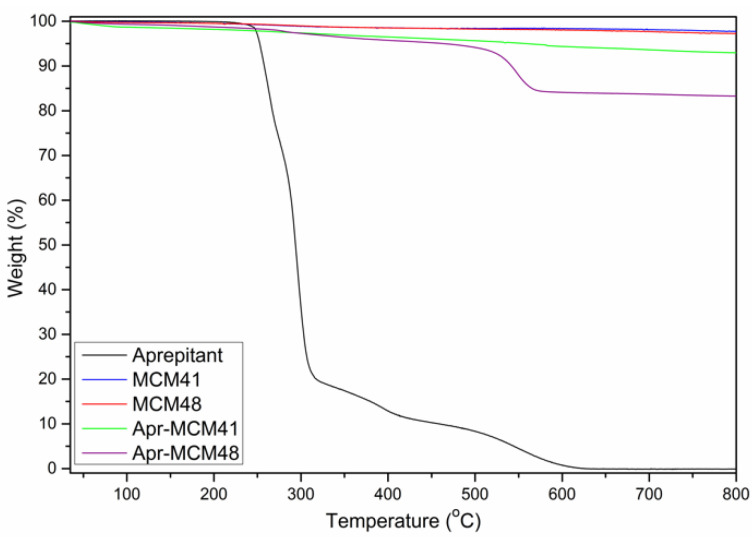
Thermogravimetric Analysis (TGA) of the pristine and drug-loaded Mesoporous Silica Nanoparticles (MSN).

The encapsulation efficiency and the drug loading studies were complemented by Thermogravimetric Analysis (TGA) studies. The amounts of the API loaded to the MSN particles, as measured by TGA (Figure 4), are summarized in Table 2. The observed mass loss in the case of aprepitant loaded nanoparticles is attributed solely to aprepitant, as there is no observable mass loss in the case of both types of MSN particles.

The data obtained from TGA analysis are in good agreement with HPLC measurements, indicating that certain quantities of the API successfully infiltrated the pores of the MSN particles, while both methods show that aprepitant infiltration is much more efficient in the case of MCM-48. In general, the relatively low amount of aprepitant loading, compared to the theoretical value, might be attributed to the particles’ narrow pore sizes, which may inhibit the infiltration of the drug molecules in the bulk of the particles. This is coupled with the hydrophobic character of the drug in contrast to the hydrophilic silica pore walls. Additionally, local sorption of the drug (e.g., on the external surface or in the mouths of the pores) may interfere with the mobility of the API’s molecules, causing a superficial accumulation and the formation of a barrier preventing further diffusion of the API into the particles’ pores. This hindered molecular diffusion—deeply into the pores—can explain the significant differences between the two MSN types. In more detail, MCM-41 pores are one-dimensional, arranged parallel to each other in a non-interconnected 2D network and thus the transfer of API molecules in the centre of the pores is heavily hampered due to the very long diffusion distances. On the other hand, the highly interconnected 3D gyroid network of MCM-48 provides much more favourable mass transfer kinetics as the diffusion distances are significantly shorter. It may also be argued that based on SEM observations, the MCM-41 particles seem to be more aggregated and/or fused than the MCM-48 ones. This agglomeration may pose additional diffusion limitations for pore infiltration as in aggregates the effective particle size (and thus diffusion length) increases significantly.

### 2.3. Powder X-ray Diffractometry (pXRD)

**Figure 5:**
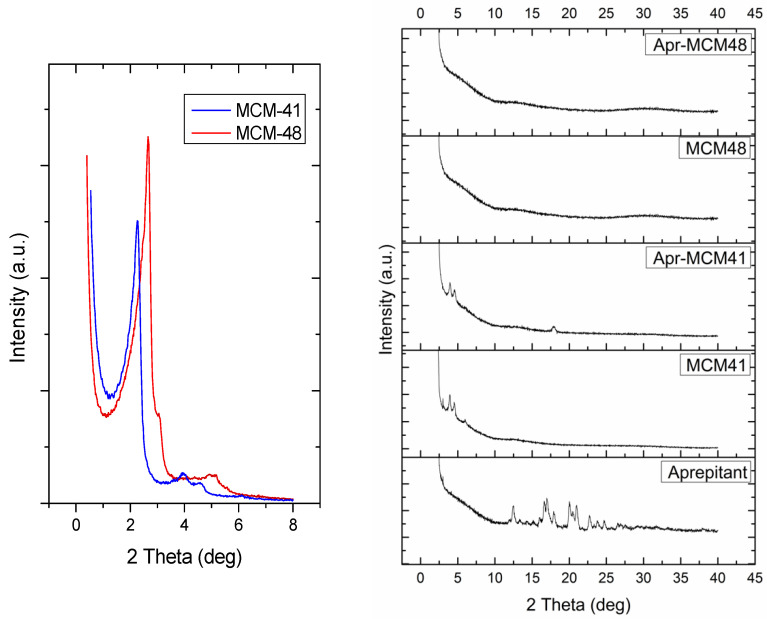
Small angle Powder X-ray Diffractometry (pXRD) patterns (**left**) of pristine MSN particles and wide angle pXRD patterns (**right**) of the samples in the presence and absence of aprepitant.

The small angle X-ray diffraction patterns of the produced MSN particles (Figure 5, left) confirmed the presence of ordered mesoporous networks in both cases. MCM-41 clearly exhibits the characteristic diffraction peaks of the 2D hexagonal lattice (*p*6mm) [27], whereas the MCM-48 pattern is typical for the cubic *Ia*3*d* symmetry [28]. The wide angle pXRD measurements of the pure API (Figure 5, right) showed the characteristic peaks of the crystalline structures I (2*θ* = 15.6°, 17.7°, and 22.21°) and II (18.3° and 21.11°) of aprepitant [17,22,23]. The absence of these peaks in Apr-MCM-48 indicates that the drug is confined inside the pores of the MSN particles in an amorphous state. On the other hand, the small peak at 2*θ* = 17.7° in the case of Apr-MCM-41 is assigned to crystalline aprepitant (structure I) and is a sign that a small quantity of the API is not pore confined but rather sorbed on the external surface of the particles.

### 2.4. Differential Scanning Calorimetry (DSC)

**Figure 6:**
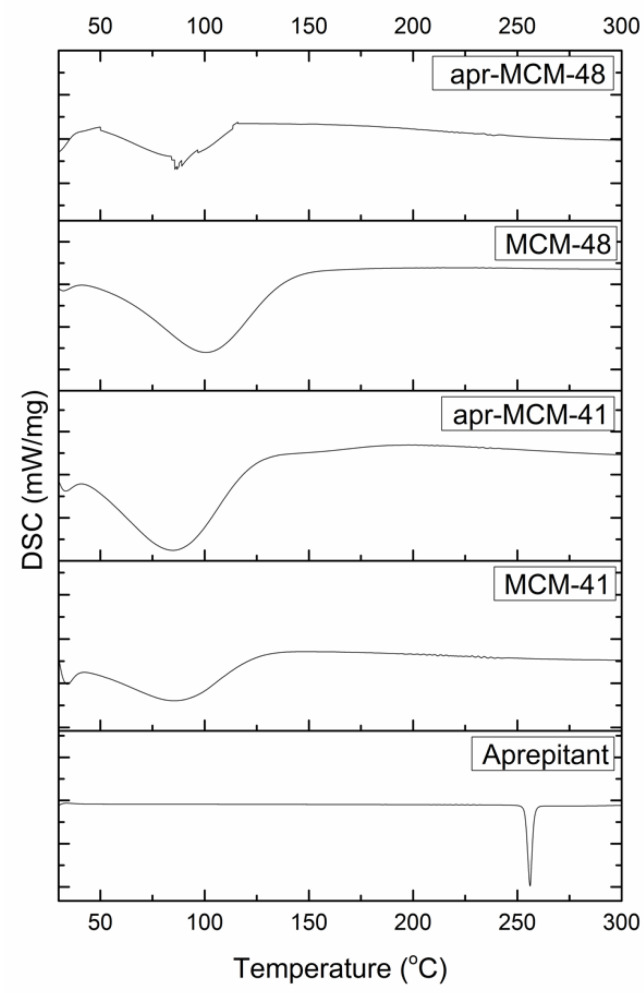
Differential Scanning Calorimetry (DSC) analysis of the pristine and drug loaded MSN particles.

Figure 6 shows the DSC thermograms obtained by the analysis of aprepitant and MSN particles (both pristine and drug loaded). The thermograms of both MSN particles exhibit a broad endothermic peak in the range of 50–150 °C, attributed to moisture desorption, while the analysis of aprepitant revealed a sharp peak at 256 °C, which is assigned to the API’s melting point. The DSC analysis of the drug loaded particles also revealed the presence of water desorption phenomena, while the melting point of the API was not detected, in agreement with the existence of an amorphous (liquid-like) sorbed state of the drug inside the pore structure of the particles as revealed by the pXRD analysis.

### 2.5. Attenuated Total Reflectance Fourier Transform Infrared Spectroscopy (ATR-FTIR)

**Figure 7:**
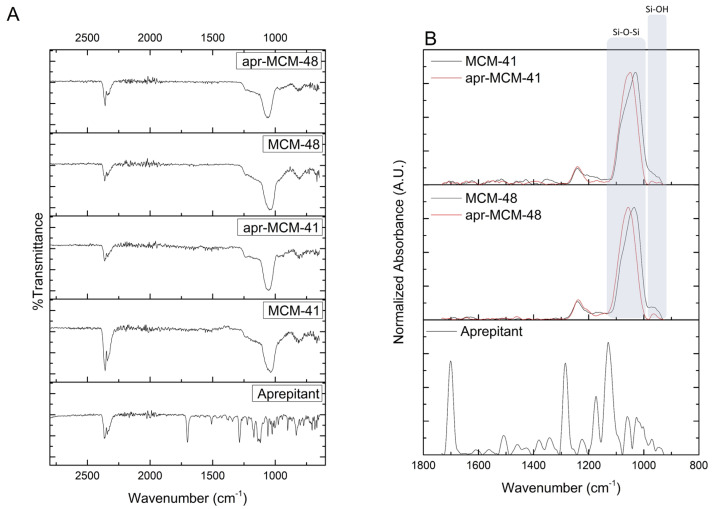
(**A**) Attenuated Total Reflectance Fourier Transform Infrared Spectroscopy (ATR-FTIR) analysis of the pristine and drug-loaded MSN particles. (**B**) Normalized absorbance ATR-FTIR spectra focused on the region between 800–1800 cm^−1^.

The vibrational features of the nanoparticles, detected by ATR-FTIR measurements, are shown in Figure 7A,B. The spectra of MCM-41 and MCM-48 show broad bands in the range 1000–1250 cm^−1^, attributed to the presence of Si-O bonds [29,30]. On the other hand, the drug’s characteristic peak at 1703 cm^−1^ is attributed to the stretching vibration of C=O bonds, while the peak at 1510 cm^−1^ is attributed to the stretching vibration of C=C bonds [23]. The peaks located at 1289, 1169, and 1069 cm^−1^ are assigned to the presence of C-O groups and the peaks around 1129, 1140, and 1117 cm^−1^ are characteristic of the C-F bond [23]. The presence of a peak at 1140 cm^−1^ indicates the polymorph type II structure of aprepitant [31].

The absence of any drug characteristic peaks in the spectra of the loaded MSN particles can be attributed to the nanoconfinement of the drug in the particles’ pore network, which leaves minuscule amounts of external, detectable aprepitant; thus, the ATR-FTIR signal is dominated mainly by the external silica surface. On the other hand, a detailed examination of the normalized absorbance ATR-FTIR spectra, focused on the region between 800–1800 cm^−1^, reveals a minor shift of the characteristic Si-O-Si peak around 1050 cm^−1^ [32,33] towards higher wavenumbers for the drug-loaded MSN particles of both types. This shift may be attributed to the possible overlapping of Si-O-Si peaks of the MSN particles with the drug’s C-O peak (1069 cm^−1^), indicating a possible interference of the C-O stretching movement of aprepitant with the asymmetric Si-O-Si stretching [32]. Additionally, the low-intensity peak around 960 cm^−1^, which is attributed to the Si-OH stretching vibrations [33], seems to diminish due to the presence of aprepitant, further indicating the possible interaction of the latter with the surface hydroxyl groups of the MSN particles.

### 2.6. Pore Analysis

The pore properties of the MSN particles before and after the loading of aprepitant were determined by N_2_ sorption measurements at 77K. The Brunauer–Emmett–Teller (BET) area of the particles was estimated by respecting the BET consistency criteria. The respective pore size distributions were determined by means of the Non-Local Density Functional Theory (NLDFT) on the basis of the N_2_-silica cylindrical pore kernel. The total pore volume of the particles was calculated by assuming that complete pore-filling occurs at p/p_0_ = 0.95, whereas the density of the adsorbate phase is equal to the density of liquid N_2_ (77K).

**Figure 8:**
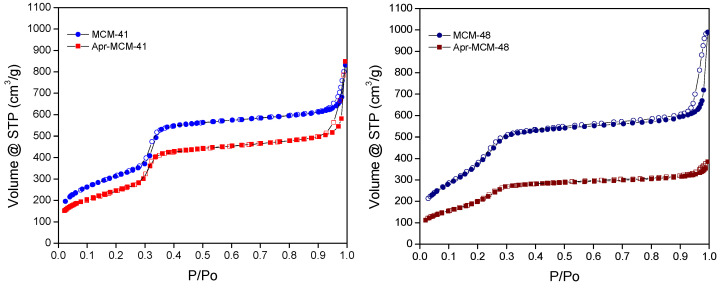
N_2_ adsorption-desorption isotherms (77K) of MCM-41 (**left**) and MCM-48 (**right**) particles in the presence and absence of aprepitant (empty symbols: desorption).

The N_2_ adsorption—desorption isotherms for the empty MCM-41 and MCM-48 particles—are depicted in Figure 8. The isotherms are of IVb type, based on IUPAC (International Union of Pure and Applied Chemistry) classification, having low-pressure abrupt condensation-evaporation steps without hysteresis, typical of ordered mesopores with sizes smaller than ~4 nm. At higher relative pressures, close to saturation, a marked increase of the amount adsorbed is revealed for both samples. This feature is coupled with desorption hysteresis and is attributed to a secondary, sphere-pack-type pore system, developed between the particles.

The isotherms of the drug-loaded samples are also shown in Figure 8. Although the amounts adsorbed are lower, the sorption isotherms are similar to the isotherms of the empty samples, implying that no structural alteration occurs upon loading. Nevertheless, it can be noticed that the decrease in amounts adsorbed is much more pronounced in the case of MCM-48, pointing to a generally more efficient loading in accordance with HPLC and TGA results. It is also noteworthy that the aforementioned “secondary” pore network (i.e., the pore space between the spherical particles) has almost disappeared in the case of the Apr-MCM-48 sample, whilst it seems even more enhanced in the case of Apr-MCM-41. The enhancement may be attributed to limited drug penetration, as explained in Section 2.2, and drug sorption on the external surface of the particles (rather than between the particles as in MCM-48). This may lead to the development of additional (extra particle) pores in contrast to Apr-MCM-48, in which the inter-particle porosity is completely blocked by aprepitant.

**Figure 9:**
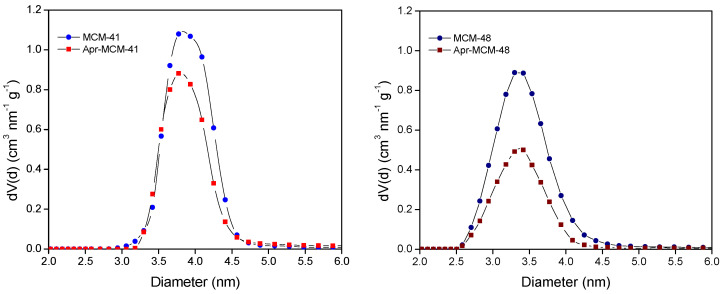
Non-Local Density Functional Theory (NLDFT) pore size distributions of pristine and aprepitant-loaded MCM-41 (**left**) and MCM-48 (**right**).

After using the NLDFT kernel for N_2_ adsorption at 77K in siliceous cylindrical pores, the mean pore size of the MCM-41 and MCM-48 particles was calculated as 3.8 nm and 3.4 nm, respectively. The pertinent size distributions are presented in Figure 9 along with the respective size distributions for the aprepitant-loaded samples. In general, the size distributions are quite sharp and typical for ordered mesoporous silicas. As in the case of the adsorption isotherms, there is a reduction of the integral pore volume (the area below the curves of Figure 9) after loading the samples with the API. Again, it is evident that the reduction is more pronounced in the case of loaded MCM-48, in agreement with HPLC and TGA results. An additional interesting feature of the pore size distributions is related with the shapes of the curves of Figure 9. In more detail, in MCM-48 the shape of the distribution curve is retained after loading, thus revealing a homogeneous filling of all the pores and therefore a homogeneous loss of pore volume. On the other hand, the size distribution of Apr-MCM-41 shows a preferential loss of larger pores, in agreement with the diffusion limited pore infiltration process that was outlined in Section 2.2.

The BET area, pore volume, and mean pore diameter (NLDFT method) values are summarized in Table 3. The empty MCM-41 and MCM-48 samples have similar BET areas (~1150 m^2^/g) and pore volumes (~1 cm^3^/g), however the pore size of MCM-41 is slightly larger compared to MCM-48, although the same porogen (CTAB) was used for their synthesis. This difference can be related to a more extensive silica network contraction of MCM-48 upon surfactant removal during calcination. As expected, the drug-loaded MCM-41 particles exhibit reduced BET area and pore volume (by approximately 21% and 18%, respectively) compared to the empty sample, indicating that a certain amount of API is entrapped within the pore structure. In a similar way, the aprepitant-loaded MCM-48 particles exhibit even larger reduction in BET area and pore volume (approximately 41% and 47%, respectively), confirming that the API is also infiltrated in the MCM-48 structure, nevertheless in a more efficient manner compared to MCM-41.

### 2.7. In Vitro Release Studies

**Figure 10:**
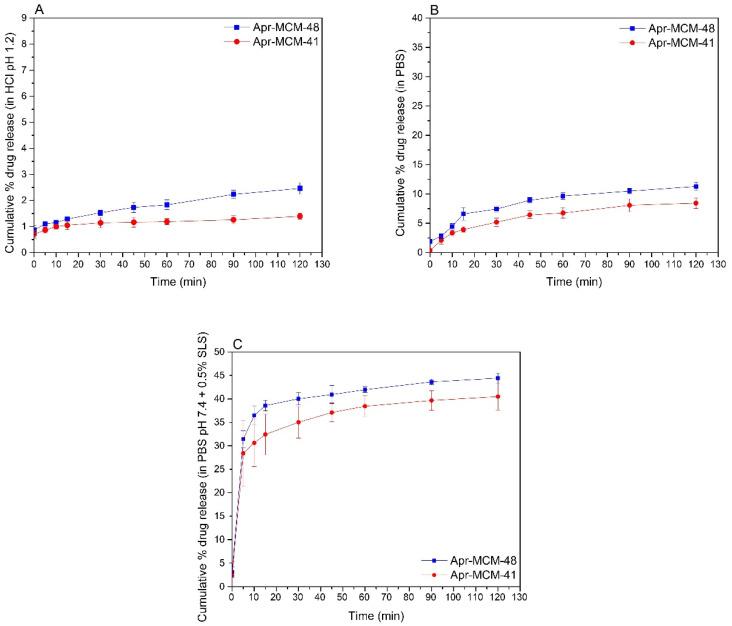
Release profile of aprepitant from MCM-41 (Apr-MCM-41) and MCM-48 (Apr-MCM-48) particles in (**A**) aqueous HCl solution pH 1.2, (**B**) in PBS pH 7.4, and (**C**) in PBS pH 7.4 in the presence of 0.5% *w/v* SLS.

The in vitro release of aprepitant from the different MSN particles was monitored in different solutions (aqueous HCl solution pH 1.2, PBS pH 7.4, and PBS pH 7.4 + 0.5% *w/v* SLS). The drug release profiles in the respective media are depicted in Figure 10A–C.

The release of aprepitant in aqueous HCl solution pH 1.2 (Figure 10A) was almost negligible and calculated to be 1.39% from MCM-41 and 2.46% from MCM-48 particles, respectively. On the other hand, the release in PBS pH 7.4 containing 0.5% *w/v* SLS was calculated as 40.48% from MCM-41 and 44.42% from MCM-48, respectively. The relatively low amount of aprepitant released in both media is attributed to the hydrophobic nature of the API that “prefers” to stay nanoconfined inside the pore structure of the particles. On the other hand, the presence of the surfactant seems to enhance the release of the API from the particles, even though the overall percentage of the released substance is considered low.

### 2.8. In Vitro Cell Studies

#### Cytocompatibility of MSN Particles and Cellular Morphology (Caco-2 Cell Culture)

**Figure 11:**
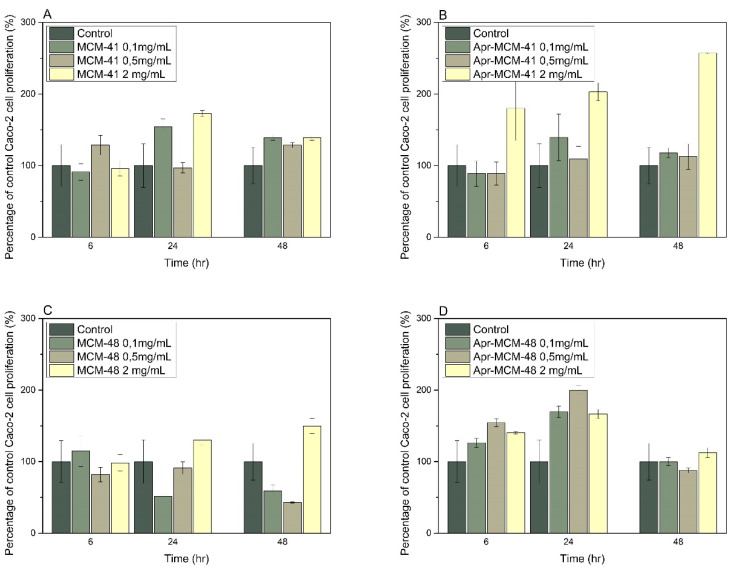
Cell growth assessment of Caco-2 cells incubated for 48 h with increasing concentrations of (**A**) MCM-41, (**B**) aprepitant-loaded MCM-41, (**C**) MCM-48, and (**D**) aprepitant-loaded MCM-48 particles.

Caco-2 cell cultures incubated for 48 h with increasing concentrations of MCM-41, aprepitant-loaded MCM-41, and aprepitant-loaded MCM-48 particles (Figure 11A,B,D, respectively) did not induce any statistically significant reduction of cell growth (t-test, *p* < 0.05), nor exhibited any increase of cell death compared to the control untreated culture. However, cell cultures incubated with MCM-48 particles (Figure 11C) demonstrated a slight inhibition of cell growth (*t*-test, *p* > 0.05) for particles concentration of 100 and 500 μg/mL, an indication that these materials might be considered potentially cytotoxic at these concentrations (ISO 10993-5). Importantly, this effect does not seem to be dose-dependent, and could be attributed to mechanical obstruction of the materials to cell proliferation potential [34,35]. The results obtained in the current study are in good agreement with previous studies where ordered mesoporous silica did not exhibit any toxicity when tested in Caco-2 cell lines [36,37,38].

## 3. Discussion

Both MCM-41 and MCM-48 particles exhibited spherical morphology (diameters 80–150 nm) and pore sizes between 3–4 nm, which is typical for these materials [1,30,36,38,39]. The pore network structure of the particles was verified by pXRD analysis and the results were in good agreement with the literature [17,22,23].

MCM-41 and MCM-48 spherical particles were loaded with aprepitant via a passive diffusion method using methanol as the most suitable solvent. The low percentage of the loaded aprepitant in all cases indicates that the incorporation of the hydrophobic drug into the rather hydrophilic pore structure of the silica particles is not favored; however, infiltration was proven to be much more effective in the case of MCM-48. This finding was related with the API diffusion limitations in the 1D non-interconnected MCM-41 channels, in contrast to the 3D interconnected MCM-48 pore network. In general, the low loading capacity of the samples observed is not uncommon for non-functionalized MSN particles [40] and highlight the necessity of appropriate modification of the mesoporous carriers (e.g., through surface functionalization) to meet the daily dosage needs of an adult, which could reach up to 125 mg per dose [19]. Nevertheless, the pore analysis of the MSN particles before and after the drug loading reveals the presence of confined aprepitant in their pore structure, while pXRD, DSC, and FTIR analysis indicate loss of API’s crystallinity due to nanoconfinement, which is key for increasing the solubility and dissolution rate of poorly soluble drugs.

The release profiles of the drug from the two different MSN particles also indicate that up to 45% aprepitant is released (within 120 min) when sink conditions are applied. According to previously reported studies [13], the dissolution of aprepitant (from commercial formulations) is positively affected by acidic environments. On the contrary, the findings of the present work show negligible release of the nanoconfined aprepitant in the aqueous HCl solution pH 1.2 (Figure 10A), as well as decreased release in PBS pH 7.4 (Figure 10B). This enhanced aprepitant retention may be linked to the FTIR analysis results presented above that show the interaction of the drug with the silica surface Si-OH groups. Additionally, the observed changes of release rate at different pH values may be connected with the variation of the degree of protonation of the Si-OH groups that can modify the wetting properties of the pore walls. On the other hand, the addition of SLS substantially promotes the release of the API from the MSN particles, in agreement with other relevant studies [3], by assisting aprepitant dissolution.

Finally, the cell viability study of Caco-2 cells incubated with MCM-41 and MCM-48 in the presence and absence of aprepitant showed that both materials do not present any significant toxicity in vitro, in good agreement with previously reported studies on similar materials [36,37,38]. All above results confirm that ordered mesoporous silica nanoparticles such as MCM-41 and MCM-48 provide a good platform for the development of effective aprepitant carriers.

## 4. Materials and Methods

### 4.1. Materials

All reagents and solvents used were of standard analytical grade and HPLC grade, respectively. Aprepitant was kindly donated by Rontis Hellas SA (Rontis Hellas SA, Athens, Greece) and used as received. Triblock copolymer EO_106_PO_70_EO_106_ (Pluronic F127), cetyltrimethylammonium bromide (CTAB), tetraethyl orthosilicate (TEOS 98%), sodium hydroxide pellets (NaOH), and ammonia 30% (NH_4_OH), used to synthesize the MSNs, were purchased from Sigma-Aldrich Inc. (St. Louis, MO, USA).

For the preparation of MCM-41 silica particles, 1.0 g of CTAB was added into a mixture of 3.5 mL of 2 M NaOH_(aq)_ solution and 480 mL of deionized water, under vigorous stirring at 80 °C. Subsequently, after dissolution of the surfactant, 5 mL of TEOS was added dropwise, and the mixture was kept under heating and stirring to create a white slurry [41]. The resulting product was obtained after filtration, washing with deionized water, drying at room temperature and calcination under air flow (80 mL/min) at 550 °C for 6h (1 °C/min).

MCM-48 silica spheres were prepared via a modified Stöber method [42]. In total, 1.0 g of CTAB and 4.0 g of Pluronic F127 were added in a mixture of 85 mL ethanol and 213 mL of 2.8 wt% NH_4_OH_(aq)_. After homogenization, 3.8 mL of TEOS were added into the solution at room temperature under vigorous stirring for 1 min. The mixture was kept under static conditions for 24 h, centrifuged (9000 rpm), washed with deionized water, dried at 40 °C, and calcined under air flow (80 mL/min) at 550 °C for 6 h (1 °C/min) [43].

### 4.2. Aprepitant Loading into MSNs

Aprepitant-loaded MSN particles were prepared by the passive diffusion method, according to (slightly modified) established procedures [39,44]. Briefly, 2 mg of MCM-41 or MCM-48 particles were added to previously prepared solutions containing 1 mg of aprepitant dissolved in 2 mL of methanol. The mixture was sonicated for 5 min using a probe sonicator (SONICS, vibracell^TM^), in an ice bath, to ensure complete particle suspension and to avoid any possible particle clustering. The suspensions were sealed to avoid solvent evaporation and left for 24 h at room temperature, under constant magnetic stirring (250 rpm). Subsequently, the samples were centrifuged at 4500 rcf for 15 min and the supernatant was collected for further analysis. The precipitated powder was rinsed three times with the respective solvent to remove any unconfined drug from the surface of the particles. Drug-loaded particles were left to dry at ambient conditions for 72 h (until achieving a constant weight). All dry samples were stored in desiccators at room temperature in the dark until further analysis.

### 4.3. HPLC Analysis of Aprepitant

Quantification of aprepitant was performed by high-performance liquid chromatography (HPLC). The system consisted of a pump (LC-10 AD VP), an auto-sampler (SIL-20A HT), and an Ultraviolet–Visible detector (SPD-10A VP, Shimadzu, Kyoto, Japan). The analytical method was adopted by previously reported studies [8]. Briefly, the stationary phase was an Ascentis C8 (15 cm × 4.6 mm, 5 μm) column and the mobile phase consisted of a mixture of acetonitrile and 0.1% orthophosphoric acid (60:40). The flow rate and the injection volume were 1.0 mL/min and 30 μL, respectively. The UV detector was set at 210 nm and the retention time of aprepitant was in the range of 5.88–6.22 min. Calibration curves were constructed using standard aprepitant solutions in the range of 0.2–100 μg/mL (R2 ≥ 0.9999).

### 4.4. Quantification of Drug Loading

The amount of aprepitant loaded into the MSN particles was quantified indirectly by analyzing the residual amount of drug in the loading solutions upon centrifugation of the dispersions (supernatant) [39,44]. Drug loading was calculated from the mass balance according to the following equation:

Drug Loaded (mg) = W_InDrug_ − W_ResDrug_ (1)

where Drug Loaded is the amount of aprepitant confined in the MSN particles in mg, W_InDrug_ is the initial amount of aprepitant in the respective loading solution in mg, and W_ResDrug_ is the residual amount of aprepitant in the supernatant in mg.

Encapsulation efficiency and Drug Loading were calculated according to Equations (2) and (3), respectively:

Encapsulation Efficiency (%) = 100 ∗ W_DrugLoaded_/W_InDrug_ (2)

where W_Drug Loaded_ is the amount of aprepitant confined in the MSN particles in mg, as it is calculated by Equation (1), and W_InDrug_ is the initial amount of aprepitant in the respective loading solution in mg.

Drug Loading (%) = 100 ∗ W_DrugLoaded_/(W_DrugLoaded_ + W_MS_) (3)

where W_Drug Loaded_ is the amount of aprepitant confined in the MSN particles in mg, as it is calculated by Equation (1), and W_MS_ is the total amount of MSN particles in the respective loading solution in mg.

Finally, the theoretical drug loading was calculated according to:

Theoretical Drug Loading (%) = 100 ∗ W_InDrug_/(W_InDrug_ + W_MS_) (4)

where W_InDrug_ is the initial amount of aprepitant and W_MS_ is the total amount of MSN particles, in the respective loading solution in mg.

### 4.5. Scanning Electron Microscopy (SEM)

The morphology of the MSN particles was studied by a scanning electron microscope equipped with a Gentle Beam mode (JSM 7401F Field Emission Microscope, JEOL Ltd.,Tokyo, Japan), under high vacuum. Conductive double-sided carbon adhesive tape (TED Pella, Redding, CA, USA) was used to mount the samples. A 2 kV accelerating voltage was used during the measurements.

### 4.6. Powder X-ray Diffracrometry (pXRD)

Small angle powder X-ray diffraction patterns of the pristine samples in powder form were recorded with the aid of “Lindemann” type glass capillaries (OD = 0.7 mm) on a Rigaku (Tokyo, Japan) R-AXIS IV Imaging Plate Detector combined with a RU-H3R Rotating Copper Anode X-ray Generator (λ = 0.154 nm). Wide angle pXRD measurements were carried out on a Bruker (Billerica, MA, USA) X-ray system (D8-Advance diffractometer equipped with a LynxEye type detector). Cu Kα radiation (λ = 0.154 nm), operated at 40 kV and 40 mA, was used. Data were collected over the 2θ range from 0° to 160° at a scanning speed of 0.35 s/step and a step size of 0.02°.

### 4.7. Differential Scanning Calorimetry (DSC)

Thermal analysis of the samples was performed by a differential scanning calorimeter (204 F1 Phoenix DSC apparatus, Netsch GmbH, Selb, Germany). Accurately weighted samples were heated, from 20 °C to 400 °C at a heating rate of 10 °C/min, under nitrogen flow (70 mL/min).

### 4.8. Thermogravimetric Analysis (TGA)

The TGA thermograms of the samples were recorded using a TG Q500 instrument (TA Instruments, New Castle, DE, USA). Samples (5 mg) were accurately weighted and heated from 25 to 900 °C, at a heating rate of 3 °C/min, under inert atmosphere.

### 4.9. ATR-FTIR Spectroscopy

The chemical structure of all formulations was studied by recording their ATR-FTIR spectra (IR Prestige-21, Shimadzu, Kyoto, Japan). The resolution of the spectrum was 4 cm^−1^ and the recorded wavenumber range was 800–4000 cm^−1^. A total of 64 spectra were averaged to reduce noise. The commercially available software IR Solutions (Shimadzu, Kyoto, Japan) was used to process the spectral data.

### 4.10. Determination of Pore Properties

The pore properties of MSN particles were analyzed by N_2_ sorption/desorption isotherms at 77 K, performed by a commercial volumetric gas adsorption system (Nova 2200e, Quantachrome Instruments, Boynton Beach, FL, USA). Overall, 50 mg of samples were outgassed (24 h at 50 °C) under high vacuum (10^−6^ mbar) prior to measurement, while ultra-pure N_2_ (99.9999%) was used. The Brunauer–Emmett–Teller (BET) area values were calculated by respecting the relevant consistency criteria. The pore size distributions were deduced based on the Non-Local Density Functional Theory (NLDFT) method by using the N_2_-silica, cylindrical geometry, adsorption branch kernel. Total pore volumes were estimated from the amount of N_2_ adsorbed at a relative pressure of 0.95.

### 4.11. In Vitro Release Studies

The release of aprepitant from the MSN particles was studied in different media, namely: Phosphate Buffer Saline (PBS; sodium chloride, 8.0 g, potassium chloride, 0.20 g, sodium phosphate dibasic, 1.44 g, potassium phosphate monobasic, 0.24 g; 1 L), pH 7.4; PBS, pH 7.4 in the presence of 0.5% *w/v* of sodium lauryl sulfate (SLS) and HCl pH 1.2. Briefly, the samples were suspended in the release medium at 37 °C and aliquots (1 mL) were withdrawn at specific time intervals (0, 5, 10, 15, 30, 60, 90 and 120 min) and immediately replaced with equal amounts of the respective medium. The experiment was conducted under constant stirring (200 rpm) at 37 °C. All collected samples were centrifuged at 4500 rcf, for 15 min, and the supernatants were filtered through 0.45 μm polyvinylidene fluoride (PVDF) filters. The amount of aprepitant that was present in the samples was quantified by HPLC. Release data were fitted with mathematical models to investigate the release mechanism of aprepitant from MS particles using the DDSolver software [45].

### 4.12. Cytotoxicity Studies

#### 4.12.1. Caco-2 Cell Cultures

Cell culture reagents Dulbecco’s Modified Eagle’s Medium (DMEM), Fetal Bovine Serum (FBS), Penicillin and Streptomycin (PS), MEM Non-Essential Amino Acids (NEAA), and trypsin, were obtained from GIBCO Thermo Fisher Scientific Inc (Waltham, MA, USA). A 3-(4,5-Dimethyl-2- thiazolyl)-2,5-diphenyl-2H-tetrazolium bromide (MTT) colorimetric assay kit was purchased from Trevigen. Caco-2 cells were grown at 37 °C in a humidified atmosphere containing 5% *v/v* CO_2_. The culture medium consisted of DMEM, supplemented with 10% *v/v* FBS, penicillin, and streptomycin S (100 μg/mL of each), and 1% *v/v* non-essential amino acids (NEAA). In stock cultures, the medium was changed every other day. Caco-2 cultures at passages 41–44 were used and cells were sub-cultured by trypsinization in tissue culture flasks.

#### 4.12.2. Cytotoxicity Assessment

The in vitro cytotoxicity effects of the MSN particles in Caco-2 cultures was assessed by counting the cell number using the improved Neubauer hemocytometer chamber and an optical microscope [34,46,47,48]. Prior to each test, the cells were harvested using trypsin (Euroclone)–ethylenediamine tetraacetic acid (EDTA, Sigma–Aldrich)–phosphate-buffered saline (PBS) solution (0.25% trypsin–0.05 mM EDTA) and diluted at a density of 10^5^ cells/mL. The cell suspension was seeded into 96-well plates (Corning Inc., Corning, NY, USA) at 100 μL/well. After cell attachment (3–4 h), the material suspensions of MCM-41, aprepitant-loaded MCM-41, MCM-48, and aprepitant-loaded MCM-48, with increasing concentration (100, 500, and 2000 μg/mL), were added to the cultures and cells were incubated for 48 h. Subsequently, cells were detached by trypsinization and the number of cells in culture was measured using the Neubauer chamber under the microscope in an attempt to evaluate the effect of the MSN particles on cell proliferation. Simultaneously, the cellular death in Caco-2 cultures was assessed using the trypan-blue dye exclusion method [34,48]. Then cell growth capacity in treated cultures was expressed as a percentage (%) of that observed in the untreated control.

### 4.13. Statistical Analysis

All data were analyzed in triplicates and Student’s t test was applied to determine statistical significance. Significance level was set at *p* < 0.05.

## 5. Conclusions

Although the two types of spherical MSN particles investigated in this study, namely MCM-41 and MCM-48, have similar pore sizes, they exhibited different sorption and release profiles of aprepitant in different release media, which may be attributed to their substantially different pore network structure. The incorporation of the hydrophobic aprepitant in the hydrophilic silica particles has proven to be much more effective in the case of MCM-48, possessing a 3D interconnected pore system that appears more appropriate to accommodate a larger drug amount, most likely by alleviating diffusion limitations that may arise in the case of the 1D non-interconnected MCM-41 channels. Quite importantly, the cytocompatibility studies show that both materials are non-toxic to human cells in vitro, demonstrating that they provide a good platform for the development of suitable carriers for poorly soluble drugs such as aprepitant. Surface modification of the MSN particles may be a suitable way for, e.g., increasing the uptake of aprepitant to meet therapeutic dose targets, however, proper attention has to be given to avoid substantial drug retention within the pores during the release process.

## Figures and Tables

**Table 1:** Drug loading and encapsulation efficiency values determined by the HPLC method.

Particle Type	Encapsulation Efficiency (%)	Drug Loading (%)
MCM-41	12.66	5.97
MCM-48	29.67	12.92

**Table 2:** Drug loading values determined by TGA analysis.

Particle Type	Drug Loading (%)
MCM-41	4.52
MCM-48	13.06

**Table 3:** Pore properties measured for the pristine and drug-loaded MSN particles.

Sample	BET Area (m^2^/g)	Pore Volume ^1^ (cm^3^/g)	Pore Diameter ^2^ (nm)
MCM-41	1145	0.98	3.8
Apr-MCM-41	905	0.80	3.8
MCM-48	1165	0.96	3.4
Apr-MCM-48	690	0.51	3.4

^1^ At p/p0 = 0.95; ^2^ Based on NLDFT analysis.
